# LINE-1 Hypomethylation in Blood and Tissue Samples as an Epigenetic Marker for Cancer Risk: A Systematic Review and Meta-Analysis

**DOI:** 10.1371/journal.pone.0109478

**Published:** 2014-10-02

**Authors:** Martina Barchitta, Annalisa Quattrocchi, Andrea Maugeri, Manlio Vinciguerra, Antonella Agodi

**Affiliations:** 1 Department GF Ingrassia, University of Catania, Catania, Italy; 2 University College London, Institute for Liver and Digestive Health, Royal Free Campus, London, United Kingdom; 3 Gastroenterology Unit, Department of Medical Sciences, IRCCS Casa Sollievo della Sofferenza, San Giovanni Rotondo, Italy; National Cancer Center, Japan

## Abstract

**Objective:**

A systematic review and a meta-analysis were carried out in order to summarize the current published studies and to evaluate LINE-1 hypomethylation in blood and other tissues as an epigenetic marker for cancer risk.

**Methods:**

A systematic literature search in the Medline database, using PubMed, was conducted for epidemiological studies, published before March 2014. The random-effects model was used to estimate weighted mean differences (MDs) with 95% Confidence Intervals (CIs). Furthermore, subgroup analyses were conducted by sample type (tissue or blood samples), cancer types, and by assays used to measure global DNA methylation levels. The Cochrane software package Review Manager 5.2 was used.

**Results:**

A total of 19 unique articles on 6107 samples (2554 from cancer patients and 3553 control samples) were included in the meta-analysis. LINE-1 methylation levels were significantly lower in cancer patients than in controls (MD: −6.40, 95% CI: −7.71, −5.09; p<0.001). The significant difference in methylation levels was confirmed in tissue samples (MD −7.55; 95% CI: −9.14, −65.95; p<0.001), but not in blood samples (MD: −0.26, 95% CI: −0.69, 0.17; p = 0.23). LINE-1 methylation levels were significantly lower in colorectal and gastric cancer patients than in controls (MD: −8.33; 95% CI: −10.56, −6.10; p<0.001 and MD: −5.75; 95% CI: −7.75, −3.74; p<0.001) whereas, no significant difference was observed for hepatocellular cancer.

**Conclusions:**

The present meta-analysis adds new evidence to the growing literature on the role of LINE-1 hypomethylation in human cancer and demonstrates that LINE-1 methylation levels were significantly lower in cancer patients than in control samples, especially in certain cancer types. This result was confirmed in tissue samples, both fresh/frozen or FFPE specimens, but not in blood. Further studies are needed to better clarify the role of LINE-1 methylation in specific subgroups, considering both cancer and sample type, and the methods of measurement.

## Introduction

Epigenetic alterations, heritable DNA modifications that do not involve changes in the DNA sequence, are associated with changes in gene expression and are important in maintaining genomic stability [1]. Among epigenetic mechanisms, DNA methylation is the most commonly studied and involved in various biological processes including cancer [2]–[5]. Global hypomethylation, an overall genome-wide reduction in DNA methylation content, is associated with genomic instability and an increased number of mutational events [6]. Genomic DNA hypomethylation is likely to result from demethylation in repetitive elements, which account for about 55% of the human genome and determine gene regulation and genomic stability [7], [8]. Long Interspersed Nucleotide Element 1 (LINE-1) and Alu repetitive elements are major constituents of interspersed DNA repeats. Due to their high occurrence throughout the genome, methylation in repetitive elements have been shown to correlate with global genomic DNA methylation content and demethylation has been associated with genome instability and chromosomal aberrations. Thus, LINE-1 and Alu have been used as global surrogate markers for estimating the genomic DNA methylation level in cancer tissues [6], [9]–[10] and in peripheral blood leukocytes [11]. LINE-1 hypomethylation was observed in several types of cancer [12]–[14] and was associated with a poor prognosis [15]. In a meta-analysis [11], global DNA hypomethylation in peripheral blood leukocytes was associated with increased cancer risk. Another meta-analysis, investigating genome-wide DNA methylation in peripheral blood DNA and cancer risk, reports a significant inverse association between genomic 5-methylcytosine levels and cancer risk, but no overall risk association using surrogates for genomic methylation, including methylation at the LINE-1 and Alu repetitive elements was found [16]. The aim of the present study was to carry out a more comprehensive systematic review and a meta-analysis in order to summarize the current published studies and to evaluate LINE-1 hypomethylation in blood and other tissues as an epigenetic marker for cancer risk.

## Methods

### Search strategy and selection criteria

A systematic literature search in the Medline database, using PubMed, was carried out for epidemiological studies, published before March 2014, investigating the association between LINE-1 hypomethylation and cancer risk. The searches were limited to studies written in English; abstracts and unpublished studies were not included. Literature search was conducted independently by two Authors. The following selection criteria were used to search articles and abstracts: (“cancer” or “tumor” or “carcinoma”) AND (“LINE-1” or “Long Interspersed Element-1” or “global”) AND (“hypomethylation” or “methylation”). Moreover, the reference lists from selected articles were checked to search for further relevant studies. No studies were excluded a priori for weakness of design or data quality. Articles were included in the quantitative analysis only if they satisfied the following criteria: (1) case-control or cohort study designs; and (2) studies that reported mean values and standard deviations (SD) of DNA methylation level in cancer patients and in control group. Furthermore exclusion criteria were as follows: (1) the study reporting only results as median of the methylation levels or through graphic display, or 95% confidence intervals (CIs) with adjusted odds ratios (OR) or relative risks for cancer risk in subjects with the lowest level of global DNA methylation (tertile, quartile or decile) compared to group with the highest level, (2) the study reporting only gene-specific DNA methylation analysis, and (3) review articles.

Where there were missing data or additional information were required, study Authors were contacted by email.

The preferred reporting items for systematic reviews and meta-analysis (PRISMA) guidelines for the conduct of meta-analysis were followed [17].

### Data collection and extraction

Two of the Authors independently reviewed all the eligible studies and abstracted the following information in a standard format: first Author's last name, year of publication, country where the study was performed, study design, cancer sites and types, sample type, experimental methods to measure global DNA methylation levels, number of cases and controls, mean values and SD of global DNA methylation levels for each group and main results.

### Statistical Analysis

All data were analyzed using the REVIEW MANAGER 5.2 software provided by the Cochrane Collaboration (http://ims.cochrane.org/revman).

The random-effects model was used to estimate weighted mean differences (MDs) with 95% CI [18] and thus, no adjustment for environmental effects was taken into account. Furthermore, subgroup analyses were conducted by sample type (tissue or blood samples), by sample source (fresh tissue or formalin-fixed, paraffin-embedded, FFPE tissue), by cancer types (colorectal, stomach, hepatocellular), and by assays used to measure global DNA methylation levels. Forest plots were generated to illustrate the study-specific effect sizes along with a 95% CI. Heterogeneity across studies, was measured using the Q-test based on the χ2 statistic, considering significant statistical heterogeneity as p<0.1. As Cochran's test only indicates the presence of heterogeneity and not its magnitude, we also reported the I^2^ statistic, which estimates the percentage of outcome variability that can be attributed to heterogeneity across studies. An I^2^ value of 0% denotes no observed heterogeneity, whereas, 25% is “low”, 50% is “moderate” and 75% is “high” heterogeneity [19]. We also estimated the between-study variance using tau-squared (τ^2^) statistic [20].

To determine the presence of publication bias, the symmetry of the funnel plots in which mean differences were plotted against their corresponding standard errors were assessed.

## Results

### Data extraction

**Figure 1:**
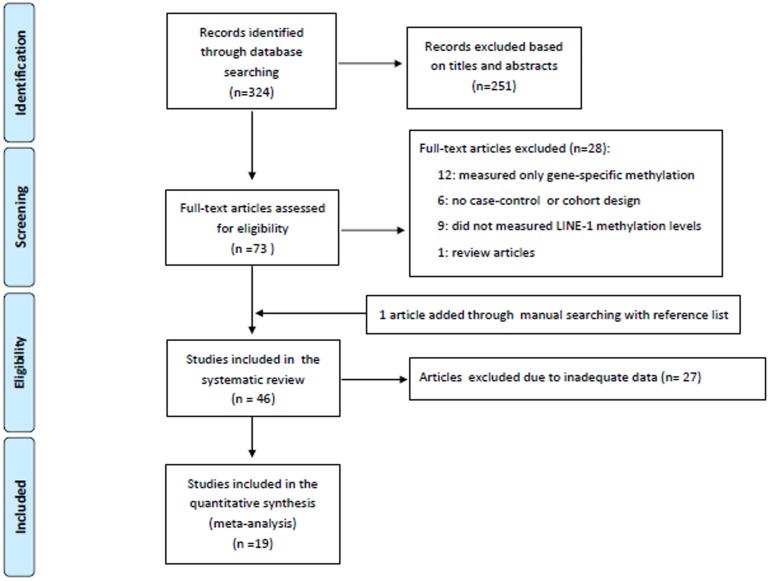
Flow diagram of study selection.

The detailed steps of the systematic review and meta-analysis process are given as a PRISMA flow chart in Figure 1. A total of 324 articles were retrieved from the database, one article was added through manual searching with reference list and thus 46 papers, published between 2004 and 2014, were included in the systematic review and summarized in Table 1 by cancer site or type.

**Table 1:** Studies included in the systematic review and in the meta-analysis.

Author (Year)	Country	Study design	Cancer type	Sample type	Assay	Number of cases/controls	Mean (SD) Cases	Mean (SD) Controls	Results
Antelo (2012)^a^*	Argentina	Retrospective	Colorectal (Early Onset)	Tissue	Pyrosequencing	185/32	56.6 (8.6)	75.5 (1.5)	Early-onset CRC had significantly lower LINE-1 methylation levels than any other group
Antelo (2012)^b^*	Argentina	Retrospective	Colorectal (Lynch Syndrome)	Tissue	Pyrosequencing	20/32	66.3 (4.5)	75.5 (1.5)	
Antelo (2012)^c^*	Argentina	Retrospective	Colorectal (Older Onset sporadic MSI-high)	Tissue	Pyrosequencing	46/32	67.1 (5.5)	75.5 (1.5)	
Antelo (2012)^d^*	Argentina	Retrospective	Colorectal (Older Onset sporadic MSS/MSI-low)	Tissue	Pyrosequencing	89/32	65.1 (6.3)	75.5 (1.5)	
Choi (2007)*	USA	Retrospective	Neuroendocrine	Tissue	Pyrosequencing	35/35	68.5 (10.0)	80.0 (7.1)	LINE-1 methylation levels were significantly lower in cancer tissues than in normal adjacent tissues
Daskalos (2009)*	UK	Retrospective	Lung	Tissue	Pyrosequencing	48/48	54.36 (10.52)	69.56 (1.1)	LINE-1 methylation levels were significantly lower in cancer tissues than in normal adjacent tissues
Estecio (2007)*	USA	Retrospective	Colorectal and various cancer cell lines	Tissue	Pyrosequencing	60/60	54.9 (1.1)	64.3 (0.5)	LINE-1 methylation levels were significantly lower in cancer tissues than in normal adjacent tissues
Hur (2014)*	Spain	Retrospective	Colorectal	Tissue	Pyrosequencing	77/77	66.2 (5.3)	75.8 (3.10)	Compared with normal adjacent mucosa, both primary cancer and metastasis tissue were significantly hypomethylated at LINE-1 elements
Iwagami (2013)*	Japan	Prospective	Esophageal	Tissue	Pyrosequencing	50/50	63.3 (12.7)	78.8 (6.2)	LINE-1 methylation levels were significantly lower in cancer tissues than in normal adjacent tissues
Lee (2008)*	Sweden	Retrospective	Thyroid	Tissue	Pyrosequencing	21/21	71.3 (2.6)	71.8 (3.4)	LINE-1 methylation changes are not observed between cancer and normal tissues
Lee (2011)*	South Korea	Retrospective	Gastric	Tissue	COBRA LINE-1	53/53	40.23 (0.92)	45.94 (1.78)	LINE-1 methylation levels were significantly lower in cancer tissues than in normal adjacent tissues
Matsunoki (2012)*	Japan	Retrospective	Colorectal	Tissue	MulticolorMethyLight Assay	48/48	63.61 (13.91)	62.54 (14)	LINE-1 methylation levels were significantly lower in cancer tissues than in normal adjacent tissues
Pavicic (2012)^a^*	Finland	Retrospective	Colorectal (Sporadic MSS)	Tissue	MS-MLPA	55/55	85 (6)	93 (2)	LINE-1 methylation levels were significantly lower in cancer tissues than in normal adjacent tissues
Pavicic (2012)^b^*	Finland	Retrospective	Colorectal (Sporadic MSI)	Tissue	MS-MLPA	52/52	87 (5)	91 (4)	LINE-1 methylation levels were significantly lower in cancer tissues than in normal adjacent tissues
Pavicic (2012)^c^*	Finland	Retrospective	Colorectal (Lynch Syndrome)	Tissue	MS-MLPA	43/43	84 (6)	90 (5)	LINE-1 methylation levels were significantly lower in cancer tissues than in normal adjacent tissues
Pavicic (2012)^d^*	Finland	Retrospective	Colorectal (FCCX)	Tissue	MS-MLPA	18/18	80 (8)	84 (6)	LINE-1 methylation levels were significantly lower in cancer tissues than in normal adjacent tissues
Pavicic (2012)^e^*	Finland	Retrospective	Gastric (Sporadic MSS)	Tissue	MS-MLPA	34/34	79 (12)	90 (5)	LINE-1 methylation levels were significantly lower in cancer tissues than in normal adjacent tissues
Pavicic (2012)^f^*	Finland	Retrospective	Gastric (Sporadic MSI)	Tissue	MS-MLPA	11/11	88 (4)	90 (4)	LINE-1 methylation levels were significantly lower in cancer tissues than in normal adjacent tissues
Pavicic (2012)^g^*	Finland	Retrospective	Gastric (Lynch Syndrome)	Tissue	MS-MLPA	13/13	86 (5)	90 (5)	LINE-1 methylation levels were significantly lower in cancer tissues than in normal adjacent tissues
Pavicic (2012)^h^*	Finland	Retrospective	Endometrial (Lynch Syndrome)	Tissue	MS-MLPA	50/50	88 (7)	90 (7)	LINE-1 methylation levels were significantly lower in cancer tissues than in normal adjacent tissues
Shigaki (2013)*	Japan	Prospective	Gastric	Tissue	Pyrosequencing	74/74	72.3 (10.1)	79.2 (5.6)	LINE-1 methylation levels were significantly lower in cancer tissues than in normal adjacent tissues
Shuangshoti (2007)*	Thailand	Retrospective	Cervix uterine	Tissue	COBRA LINE-1	7/15	35.63 (7.32)	40.6 (8.86)	LINE-1 methylation levels were significantly lower in cancer patients than in healthy subjects
Subbalekha (2008)*	Thailand	Retrospective	Head and Neck	Oral rinses	COBRA LINE-1	38/37	37.53 (2.61)	41.78 (2.84)	LINE-1 methylation levels were significantly lower in cancer patients than in healthy subjects
Cash (2012)*	China	Retrospective	Bladder	Blood	Pyrosequencing	510/528	81.86 (1.82)	81.96 (1.89)	LINE-1 methylation were comparable in cases and controls
Liao (2011)*	Central and eastern Europe	Retrospective	Renal cell	Blood	Pyrosequencing	328/654	82.13 (1.86)	81.74 (1.98)	LINE-1 methylation levels were significantly higher in cancer patients than in healthy subjects
Mirabello (2010)*	USA	Retrospective	Testicular	Blood	Pyrosequencing	152/255	79.1 (0.177)	79.3 (0.128)	There was no significant difference between LINE-1 methylation levels in cases and controls
Ramzy (2011)*	Egypt	Retrospective	Hepatocellular	Blood	COBRA LINE-1	50/10	41.86 (10.06)	54.00 (7.82)	LINE-1 methylation levels were significantly lower in cancer patients than in healthy subjects
Tangkijvanich (2007)*	Thailand	Retrospective	Hepatocellular	Blood	COBRA LINE-1	85/30	46.83 (7.74)	53.45 (4.29)	LINE-1 methylation levels were significantly lower in cancer patients than in healthy subjects
Wu (2012)*	Taiwan	Retrospective	Hepatocellular	Blood	Pyrosequencing	302/1250	76.2 (2.2)	76.2 (2.1)	There was no significant difference between LINE-1 methylation levels in cases and controls
Baba (2010)	USA	Prospective	Colorectal	Tissue	Pyrosequencing	869/NA			Tumor LINE-1 methylation data indicate enormous epigenomic diversity of individual colorectal cancers
Bae (2012)	South Korea	Prospective	Gastric	Tissue	Pyrosequencing	447(two sets of 249 and 198)/NA			LINE-1 hypomethylation is an early event in carcinogenesis and it may be a prognostic indicator independent of cancer stage
Bollati (2009)	Italy	Retrospective	Multiple Myeloma	Bone marrow aspirates	Pyrosequencing	76/11			Cases showed a decrease of LINE-1 methylation levels compared to controls
Chalitchagorn (2004)	Thailand	Retrospective	Gastric	Blood	COBRA LINE-1	17/17			Cases showed a decrease of LINE-1 methylation levels compared to controls
Choi (2009)	USA	Retrospective	Breast	Blood	Pyrosequencing	19/18			There was no significant difference between LINE-1 methylation levels in cases and controls
Dammann (2010)	Germany	Retrospective	Ovarian	Tissue	QUBRA	22/NA			High prevalence of LINE1 hypomethylation throughout all tumor stages
Di (2011)	China	Retrospective	Hepatocellular	Blood	Pyrosequencing	315/356			Hypomethylation lead to a significant 2.6-fold increased risk for HCC
Fabris (2011)	Italy	Retrospective	Chronic lymphocytic leukemia	Blood	Pyrosequencing	77/7			LINE-1 methylation levels were significantly lower in cancer patients than in healthy subjects
Gao (2012)	China	Retrospective	Gastric	Blood	Pyrosequencing	192/384			There was no significant difference between LINE-1 methylation levels in cases and controls
Gao (2013)	China	Prospective	Hepatocellular	Tissue	Sequencing and Real-time qPCR	243/48			Hypomethylation of LINE-1 was associated with tumour progression, larger tumour size, higher recurrence rates, worse tumour stage and poor tumour differentiation
Geli (2008)	Sweden	Retrospective	Pheochromocytoma and Paraganglioma	Tissue	Pyrosequencing	55/NA			Cases showed a decrease LINE-1 methylation levels compared with controls
Hsiung (2007)	USA	Retrospective	Head and Neck	Blood	COBRA LINE-1	278/526			The median methylation level in controls was slightly but significantly higher than the median level in cases. Hypomethylation lead to a significant 1.6-fold increased risk for disease
Hou (2010)	Poland	Retrospective	Gastric	Blood	Pyrosequencing	302/421			Cancer risk was highest among those with lowest level of methylation in LINE-1 relative to those with the highest levels, although the trends were not statistically significant
Igarashi (2010)	Japan	Retrospective	GIST	Tissue	Pyrosequencing	106/NA			LINE-1 hypomethylation correlates significantly with the aggressiveness of tumors and it could be a useful marker for risk assessment
Kreimer (2013)	Germany	Retrospective	Bladder	Tissue	Pyrosequencing	23/12			LINE-1 methylation was significantly decreased in cancers compared to normal tissues with striking differences in their percent median values
Ogino (2008)	USA	Prospective	Colorectal	Tissue	Pyrosequencing	643/NA			LINE-1 hypomethylation was linearly associated with a statistically significant increase in cancer – specific mortality
Phokaew (2008)	Thailand	Retrospective	Head and Neck	Tissue	COBRA LINE-1	11/12			LINE-1 methylation level at each locus is different, it can be influenced differentially depending on where the particular sequences are located in the genome
Pobsook (2011)	Thailand	Retrospective	Head and Neck	Various	COBRA LINE-1	90/114			LINE-1 partial methylation represents hypomethylation in normal cells but hypermethylation in cancer cells
Saito (2010)	Japan	Retrospective	Lung	Tissue	Real-time PCR	379/333			LINE-1 methylation levels were significantly lower in cancer patients than in healthy subjects
Sigalotti (2011)	Italy	Retrospective	Melanoma	Tissue	Pyrosequencing	42/4			LINE-1 methylation is identified as a molecular marker of prognosis
Sunami (2011)	USA	Retrospective	Colorectal	Tissue	AQAMA-PCR	117/117			LINE-1 hypomethylation was significantly greater in adenoma tissue compared to its contiguous normal epithelium and cancer mesenchymal tissue
Trankenschuh (2010)	Germany	Retrospective	FLC	Tissue	Pyrosequencing	25/15			No evidence of global hypomethylation was found
Van Hoesel (2012)	USA	Prospective	Breast	Tissue	AQAMA-PCR	129/109			LINE-1 hypomethylation is a prognostic biomarker of poor outcome
Wilhelm 2010	USA	Retrospective	Bladder	Blood	Pyrosequencing	285/465			Being in the lowest LINE1 methylation decile was associated with a significant 1.8-fold increased risk of cancer
Wolff (2010)	USA	Retrospective	Bladder	Tissue	Pyrosequencing	113/63			Cases showed a decrease LINE-1 methylation levels compared with controls
Yegnasubramanian (2008)	USA	Retrospective	Prostate	Tissue	COMPARE	76/24			Cases showed a decrease LINE-1 methylation levels compared with controls
Zhu (2011)	USA	Retrospective	Various	Blood	Pyrosequencing	205/487			Individuals with lowest LINE-1 methylation levels had a significant 4.4-fold increased incidence of lung cancer. No significant associations were observed for other tumors

(*) Studies included in the meta-analysis (N = 19).

AQAMA-PCR: Absolute Quantitative Assessment Of Methylated Alleles PCR, COBRA LINE-1: Combined Bisulfite Restriction Analysis LINE-1, COMPARE: Combination Of Methylated DNA Precipitation And Restriction Enzyme digestion, CRC: Colorectal Cancer, FCCX: Familial Colorectal Cancer type X, FLC: Fibrolamellar Carcinoma, GIST: Gastrointestinal Stromal Tumors, LINE-1: Long Interspersed Nucleotide Element 1, MSI: MicroSatellite Instable, MS-MLPA: Methylation-Specific Multiplex Ligation-dependent Probe Amplification, MSS: MicroSatellite Stable, QUBRA: Quantitative Bisulfite Restriction Analysis.

### Data characteristics and quality assessment

A total of 18 studies were from Asian countries (40%), 13 from European countries (28%), 13 from USA (28%) and 1 from Argentina and from Egypt (2%, each).

Thirty-eight retrospective longitudinal studies compared LINE-1 methylation levels between cancer patients and healthy subjects or normal adjacent tissues in cancer patients. Eight prospective longitudinal studies analysed LINE-1 methylation levels in cohorts of cancer patients, in relation to the life expectancy, the outcome of the disease or the malignancy of the tumor, identifying the role of LINE-1 hypomethylation as a biomarker of poor prognosis in cancer patients [15], [21]–[27].

In 41 studies LINE-1 methylation levels were evaluated both in tumor and in healthy controls tissues, and in the remaining 5 studies only in cancer patients.

Overall, the studies detected LINE-1 methylation levels in 15332 samples: 8103 from cancer patients (4679 tissue samples, 3276 blood samples ,72 oral rinses and 76 bone marrow plasma cells) and 7136 control samples (6277 from healthy subjects and 859 from normal adjacent tissues in cancer patients).

Regarding the experimental methods to measure LINE-1 methylation levels, the “gold standard” method, used in 63% of studies, was the pyrosequencing of bisulphite converted DNA. Furthermore, 9 studies used combined bisulphite restriction analysis of LINE-1 (COBRA LINE-1) and 8 studies used other methods, i.e. sequencing, real-time PCR, AQAMA PCR, COMPARE methylation assay, MulticolorMethyLight Assay and MS-MLPA.

The most frequent tumor type in study was colorectal cancer analyzed in eight studies [15], [21], [28]–[33], followed by seven studies that evaluated methylation level in gastric cancer [23], [27], [32], [34]–[37], five in hepatocellular carcinoma [25], [38]–[41], four in bladder cancer [1], [14], [42], [43] and head and neck carcinoma [10], [44]–[46], two in lung cancer [47], [48] and breast cancer [24], [49], and single studies assessed methylation levels in renal cell cancer [50], prostate cancer [51], neuroendocrine tumor [52], ovarian cancer [53], thyroid cancer [54], esophageal cancer [26], cervix cancer [55], endometrial cancer [32], skin melanoma [22], testicular cancer [56], leukemia [57], multiple myeloma [58], paraganglioma [59], fibrolamellar carcinoma [60] and gastrointestinal [61]. Four studies evaluated methylation level in several cancer sites [13], [28], [29], [32]. With regard to the assay method, pyrosequencing was used in 29 studies, followed by COBRA in 9 studies, Real-Time PCR and AQAMA-PCR in 2 studies. MulticolorMethyLight Assay, MS-MLP, COMPARE and QUBRA were adopted in single study each.

### Meta-analysis

Of the 46 selected papers, 14 reported means and SD of DNA methylation levels. In addition, means and SDs were independently calculated using data from 2 articles and, among Authors contacted for missing data, 3 responded to the email requests and data were added in the analysis [30], [50], [56]. Thus, 19 unique articles were included in the quantitative analysis. Furthermore, two papers by Antelo et al. [28] and by Pavicic et al. [32], reported data from different cancer types and thus, they were separated in 4 and 8 sub-studies, respectively (Table 1).

A total of 6107 samples were included in the analysis: 2554 from cancer patients (1127 tissue samples and 1427 blood samples) and 3553 control samples (2811 from healthy subjects and 742 from normal adjacent tissues in cancer patients).

**Figure 2:**
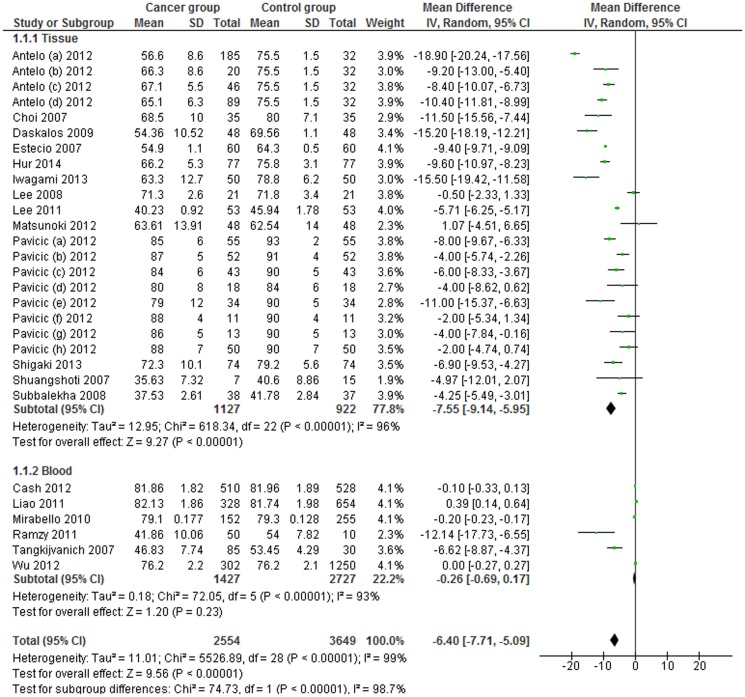
Forest plot of the mean difference of LINE-1 methylation levels between cancer and control groups in tissue and blood samples. Subgroup analysis based on sample type.

LINE-1 methylation levels were significantly lower in cancer patients than in control samples (MD: −6.40, 95% CI: −7.71, −5.09; p<0.001). However, heterogeneity between studies was significantly high (I^2^ = 99%) (**Figure 2**), thus, subgroup analysis based on sample type (tissue or blood samples) was performed. The significant difference in methylation levels was confirmed in tissue samples (MD −7.55; 95% CI: −9.14, −65.95; p<0.001), but not in blood samples (MD: −0.26, 95% CI: −0.69, 0.17; p = 0.23).

**Figure 3:**
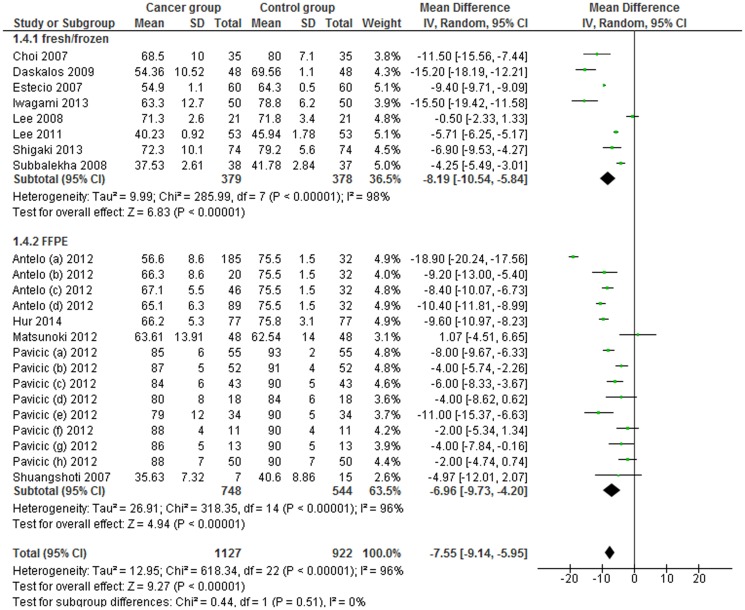
Forest plot of the mean difference of LINE-1 methylation levels between cancer and control groups in tissue samples. Subgroups analysis based on sample source.

A subgroup analysis by sample source was conducted. LINE-1 methylation levels were significantly lower in cancer patients than in control samples in fresh and/or frozen tissue (MD −8.19; 95% CI: −10.54, −5.84; p<0.001) and in FFPE tissue (MD: −6.96; 95% CI: −9.73, −4.20; p<0.001). Heterogeneity between studies, in the two subgroups was significantly high (I^2^ = 98% and 96% respectively) (**Figure 3**).

**Figure 4:**
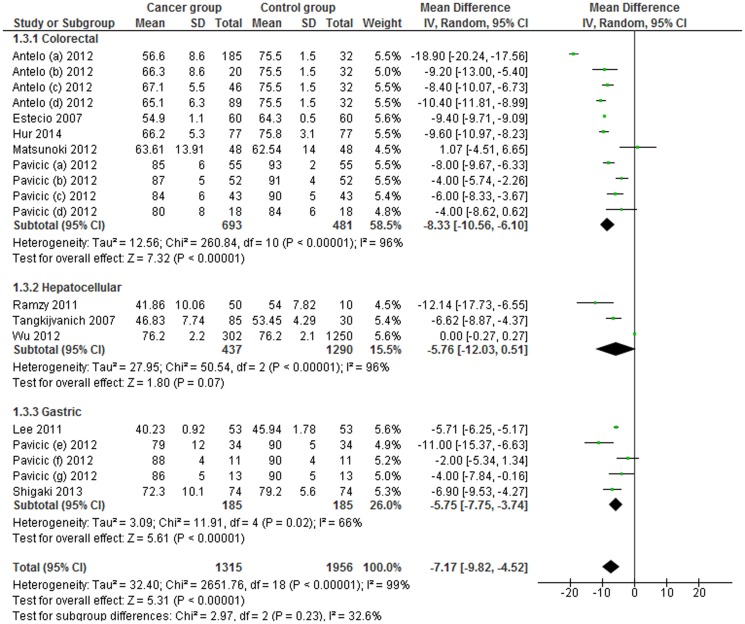
Forest plot of the mean difference of LINE-1 methylation levels between cancer and control groups. Subgroups analysis based on cancer type.

Furthermore, a subgroup analysis by specific cancer types, for colorectal, hepatocellular and gastric cancer, was conducted. LINE-1 methylation levels were significantly lower in colorectal and gastric cancer patients than in control samples (MD: −8.33; 95% CI: −10.56, −6.10; p<0.001 and MD: −5.75; 95% CI: −7.75, −3.74; p<0.001). No difference of LINE-1 methylation levels in blood leukocytes was observed for hepatocellular cancer (MD: −5.76; 95% CI: −12.03, +0.51; p = 0.23). Heterogeneity between studies, in colorectal and hepatocellular subgroups was significantly high (I^2^ = 96%), and moderately high in the gastric subgroups (I^2^ = 66%) (**Figure 4**).

**Figure 5:**
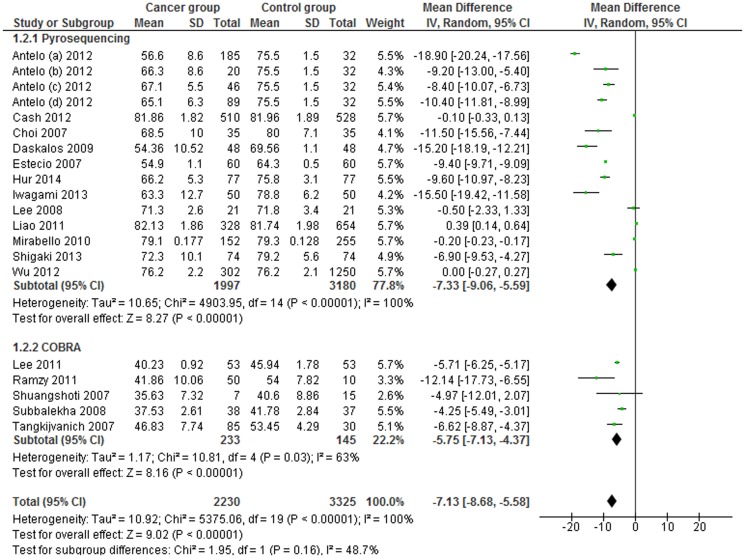
Forest plot of the mean difference of LINE-1 methylation levels between cancer and control groups. Subgroups analysis based on method.

A subgroup analysis by assays used to measure the methylation levels, and particularly, between the two commonly used techniques, pyrosequencing and COBRA LINE-1, was performed. The MDs for *pyrosequencing* and *COBRA LINE-1* subgroups were -7.33 (95% CI: −9.06, −5.59; p<0.001) and −5.75 (95% CI: −7.13, −4.37; p = 0.03), respectively. Heterogeneity between studies and in the *pyrosequencing* subgroup was significantly high (I^2^ = 100%), and moderately high in the *COBRA* subgroup (I^2^ = 63%) (**Figure 5**).

**Figure 6:**
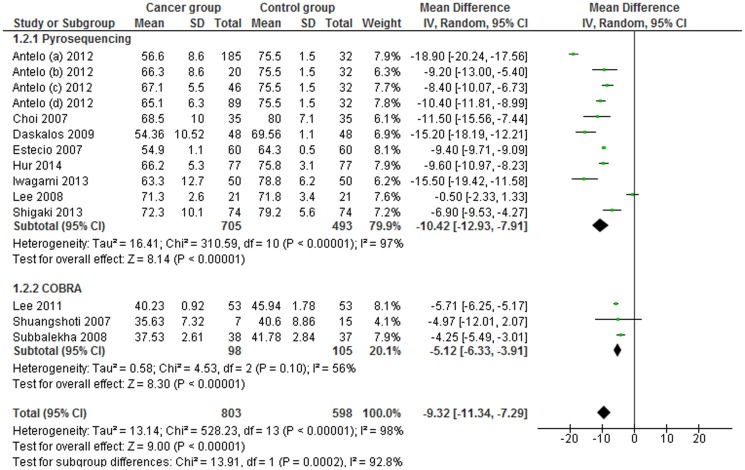
Forest plot of the mean difference of LINE-1 methylation levels between cancer and control groups in tissue samples. Subgroups analysis based on method.

A subgroup analysis by sample type, particularly tissue samples, and assay method was conducted. The MDs in the subgroups of studies which detected LINE-1 methylation levels in tissue samples through pyrosequencing and COBRA LINE-1, were −10.42 (95% CI: −12.93, −7.91; p<0.001) and −5.12 (95% CI: −6.33, −3.91; p = 0.10), respectively. Heterogeneity between studies and in the *pyrosequencing* subgroup was significantly high (I^2^ = 97%), moderately high in *COBRA LINE-1* subgroup (I^2^ = 56%) (**Figure 6**). Stratification among studies which detected LINE-1 methylation in blood samples was not performed due to the paucity of studies.

**Figure 7:**
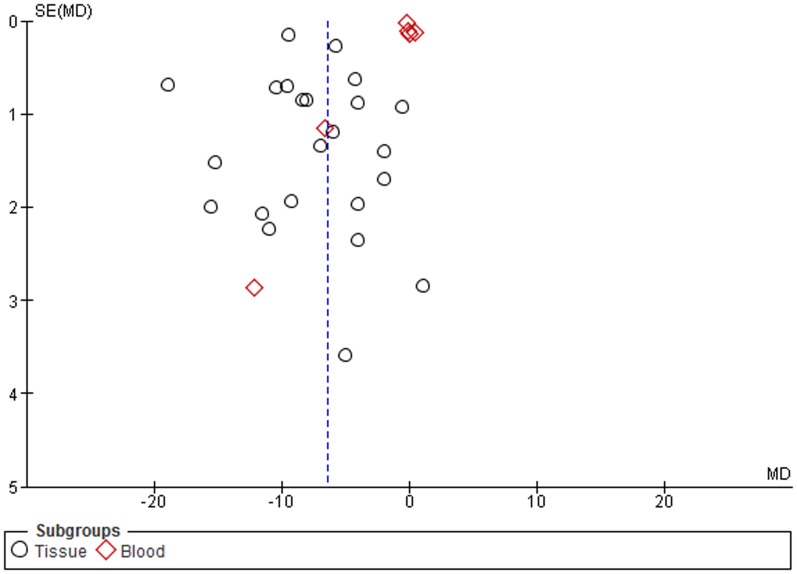
Funnel plot. Subgroup analysis based on sample type.

The funnel plots indicate small to moderate asymmetry, suggesting that publication bias cannot be completely excluded as a factor of influence on the present meta-analysis (**Figures 7** **–** **12**).

**Figure 8:**
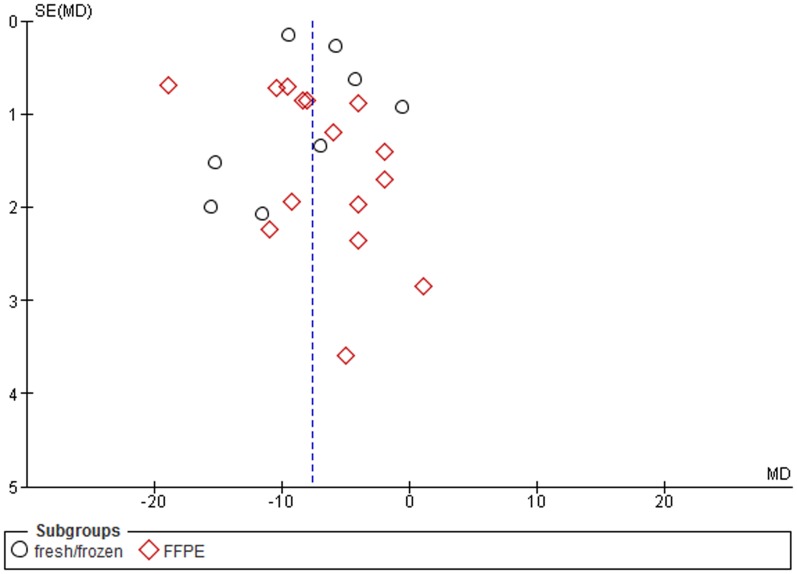
Funnel plot. Subgroup analysis based on tissue specimen types. SE, standard error, MD, mean difference.

**Figure 9:**
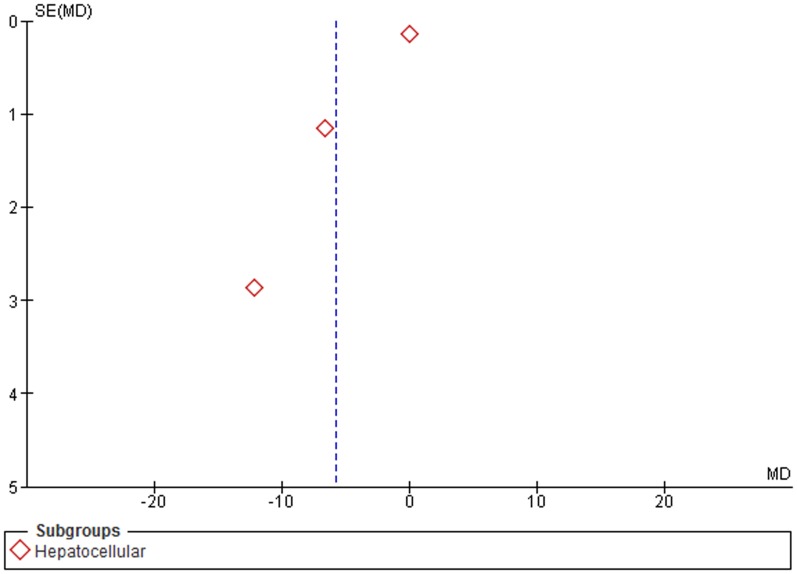
Funnel plot. Subgroup analysis based on cancer type in blood samples.

**Figure 10:**
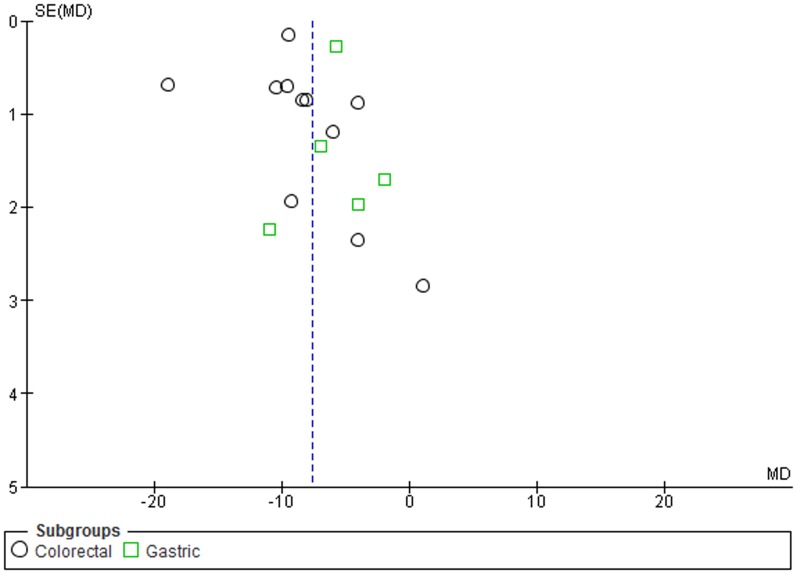
Funnel plot. Subgroup analysis based on cancer type in tissue samples.

**Figure 11:**
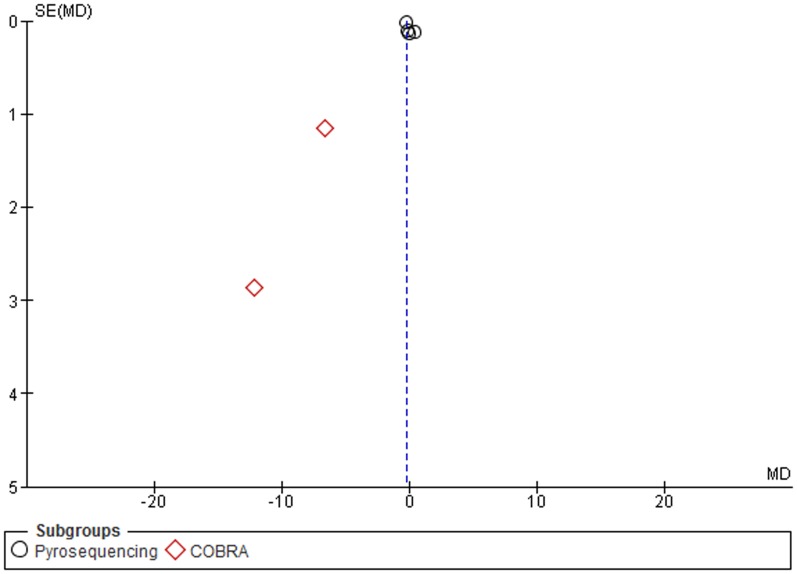
Funnel plot. Subgroup analysis based on detection method in blood samples.

**Figure 12:**
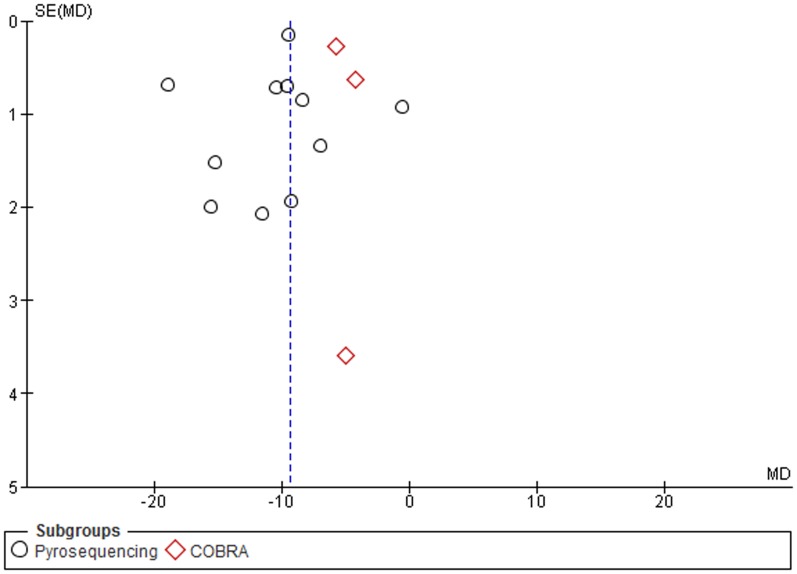
Funnel plot. Subgroup analysis based on detection method in tissue samples.

## Discussion

The low level of DNA methylation in tumors compared with DNA methylation level in their normal-tissue counterparts was one of the first epigenetic alterations to be found in human cancer [62]. The loss of methylation is mainly due to hypomethylation of repetitive DNA sequences and LINE-1 elements are typically heavily methylated in normal tissues, while LINE-1 hypomethylation has been reported in cancer tissues. Furthermore, Liao et al. [50] reported that LINE-1 methylation levels, measured in leukocyte DNA, were significantly higher in renal cancer patients than in healthy subjects.

Two recent meta-analyses were conducted in order to estimate overall cancer risk according to global DNA hypomethylation in blood leukocytes. The meta-analysis by Woo and Kim [11] reports that global DNA hypomethylation of blood leukocytes was associated with increased cancer risk, although the association varied by the experimental methods used (% 5- methylcytosine method, LINE-1 with pyrosequencing and methyl acceptance assay), the region of DNA targeted and the cancer type. An updated meta-analysis performed by Brennan and Flanagan [16] indicates a significant inverse association between genomic 5-methylcytosine levels and cancer risk (OR  = 3.65; 1.20–6.09), but no overall risk association for studies using surrogates for genomic methylation, including methylation at the LINE-1 repetitive element (OR  = 1.24; 0.76–1.72). Notably, the previous two meta-analyses included studies reporting association analysis between blood methylation levels and cancer risk but did not evaluate studies reporting differences in mean methylation levels in blood and in other tissues. The present meta-analysis of recent reports was conducted including studies reporting methylation levels in blood and in other tissues. This meta-analysis concerned 19 unique articles, but since two articles comprised more than one study conducted on different patient populations, altogether there were 29 non-unique studies included. On a total of 2554 samples from cancer patients and 3553 control samples, this meta-analysis reports that mean methylation levels in cancer patients were significantly 6.4% lower than in control samples.

The association between cancer risk and global DNA methylation has been mostly investigated in blood samples, because harvesting tumor tissue is invasive and cannot be routinely performed. However, several studies have reported that methylation of repetitive elements is tissue specific, most variable in tumor tissue, and is not correlated between tumor and blood [63]–[65]. Consistently, evidence reveals that genomic hypomethylation in tumor and normal adjacent tissue of bladder and colon cancer was not detectable in blood [43], [66], suggesting that hypomethylation is restricted to the disease affected tissue. Interestingly, in the present meta-analysis the significant difference in mean methylation levels was confirmed only in tissue samples, both fresh/frozen or FFPE specimens, but not in blood samples. Furthermore, the meta-analysis provided sufficient evidence that LINE-1 hypomethylation, significantly increases in colorectal and gastric cancer. On the contrary, no overall association was found for hepatocellular carcinoma. Notably, all studies focusing on colorectal and gastric tumors evaluated LINE-1 methylation in tissue samples, while all the included studies on hepatocellular carcinoma investigated the association only in blood leukocyte samples. Global DNA methylation can be measured by direct and indirect quantification assays. Although the measurement of percentages of 5- methylcytosine to estimate global DNA methylation contents are highly quantitative and reproducible, they require high amount of DNA and are not suitable for large epidemiological studies. Pyrosequencing with bisulfite-treated DNA, the "gold standard" for DNA methylation analysis [67], [68], is a high-throughput and accurate method currently available to measure LINE-1 methylation as surrogate marker for global DNA hypomethylation. However, LINE-1 methylation levels can vary depending on the target CpG sequence detected [69], representing an important factor in the association study with cancer risk. In the present meta-analysis, considering the two most frequently used detection methods (pyrosequencing and COBRA LINE-1) both subgroups report significantly lower LINE-1 methylation levels in cancer patients than in control samples, although heterogeneity between studies was significantly high in the *pyrosequencing* subgroup and moderately high in the *COBRA* subgroup.

The main limitations of this meta-analysis are the small number of studies included (n = 19) and the high heterogeneity across studies. Although a random effects model was performed, in order to take into account the high heterogeneity, the pooled estimates should be interpreted with caution. To overcome this issue, pooled estimates were calculated in more homogeneous subsets of studies (subgroups analysis). In addition, the possible existence of a publication bias was considered. Examination of funnel plots showed small to moderate asymmetry, suggesting that publication bias cannot be completely excluded and may have had at least a moderate impact on the true effect size estimates. In fact, some data, such as conference abstracts, non-English literature, unpublished data and other inconsistent reports according to our selection criteria were excluded. Furthermore, methylation-risk association tend only to be reported if it reveals statistically significant results, and if the authors deem analysis appropriate [16].

Moreover, since most studies (83%) had a case-control design large cohort studies are needed in order to clarify if global hypomethylation is an early cancer-causing aberration or an effect of carcinogenesis [11].

In conclusion, the present meta-analysis adds new evidence to the growing literature on the role of LINE-1 hypomethylation in human cancer and shows that LINE-1 methylation levels were significantly lower in cancer patients than in controls, especially for certain cancer types. This result was confirmed in tissue samples but not in blood. Further studies are needed to better clarify the role of LINE-1 methylation in specific subgroups, considering both the cancer and sample type, and the methods of measurement.

## Supporting Information

Checklist S1 **PRISMA Checklist.**
(DOC)

Checklist S2 **Meta-analysis on Genetic Association Studies Checklist.**
(DOC)

## References

[1] Cash H, Tao L, Yuan JM, Marsit C, Houseman E, et al (2012) LINE-1 hypomethylation is associated with bladder cancer risk among non-smoking Chinese. Int J Cancer 130: 1151–1159. 21445976 10.1002/ijc.26098 PMC3208798

[2] Laird PW, Jaenisch R (1994) DNA methylation and cancer. Hum Mol Genet 3: 1487–1496. 7849743 10.1093/hmg/3.suppl_1.1487

[3] Jones PA (1996) DNA methylation errors and cancer. Cancer Res 56: 2463–2467. 8653676

[4] Liu L, Wylie RC, Andrews LG, Tollefsbol TO (2003) Aging, cancer and nutrition: the DNA methylation connection. Mech Ageing Dev 124: 989–998. 14659588 10.1016/j.mad.2003.08.001

[5] Davis CD, Uthus EO (2004) DNA methylation, cancer susceptibility, and nutrient interactions. Exp Biol Med 229: 988–995. 10.1177/153537020422901002 15522834

[6] Chen RZ, Pettersson U, Beard C, Jackson-Grusby L, Jaenisch R (1998) DNA hypomethylation leads to elevated mutation rates. Nature 395: 89–93. 9738504 10.1038/25779

[7] Lander ES, Linton LM, Birren B, Nusbaum C, Zody MC, et al (2001) Initial sequencing and analysis of the human genome. Nature 409: 860–921. 11237011 10.1038/35057062

[8] Rodriguez J, Frigola J, Vendrell E, Risques RA, Fraga MF, et al (2006) Chromosomal instability correlates with genomewide DNA demethylation in human primary colorectal cancers. Cancer Res 66: 8462–9468. 16951157 10.1158/0008-5472.CAN-06-0293

[9] Agnelli L, Bicciato S, Mattioli M, Fabris S, Intini D, et al (2005) Molecular classification of multiple myeloma: a distinct transcriptional profile characterizes patients expressing CCND1 and negative for 14q32 translocations. J Clin Oncol 23: 7296–7306. 16129847 10.1200/JCO.2005.01.3870

[10] Fabris S, Ronchetti D, Agnelli L, Baldini L, Morabito F, et al (2007) Transcriptional features of multiple myeloma patients with chromosome 1q gain. Leukemia 21: 1113–1116. 17315022 10.1038/sj.leu.2404616

[11] Woo HD, Kim J (2012) Global DNA Hypomethylation in Peripheral Blood Leukocytes as a Biomarker for Cancer Risk: A Meta-Analysis. PLoS ONE 7: e34615. 22509334 10.1371/journal.pone.0034615 PMC3324531

[12] Hsiung D, Marsit C, Houseman E, Eddy K, Furniss C, et al (2007) Global DNA Methylation Level in Whole Blood as a Biomarker in Head and Neck Squamous Cell Carcinoma. Cancer Epidemiol Biomarkers Prev 16: 108–114. 17220338 10.1158/1055-9965.EPI-06-0636

[13] Zhu ZZ, Sparrow D, Hou L, Tarantini L, Bollati V, et al (2011) Repetitive element hypomethylation in blood leukocyte DNA and cancer incidence, prevalence and mortality in elderly individuals: the Normative Aging Study. Cancer Causes Control 22: 437–447. 21188491 10.1007/s10552-010-9715-2 PMC3752839

[14] Wilhelm C, Kelsey K, Butler R, Plaza S, Gagne L, et al (2010) Implications of LINE1 Methylation for Bladder Cancer Risk in Women. Clin Cancer Res 16: 1682–1689. 20179218 10.1158/1078-0432.CCR-09-2983 PMC2831156

[15] Baba Y, Huttenhower C, Nosho K, Tanaka N, Shina K, et al (2010) Epigenomic diversity of colorectal cancer indicated by LINE-1 methylation in a database of 869 tumors. Molecular Cancer 9: 125. 20507599 10.1186/1476-4598-9-125 PMC2892454

[16] Brennan K, Flanagan JM (2012) Is there a link between genome-wide hypomethylation in blood and cancer risk? Cancer Prev Res (Phila) 5: 1345–1357. 23135621 10.1158/1940-6207.CAPR-12-0316

[17] Moher D, Liberati A, Tetzlaff J, Altman DG (2010) PRISMA Group (2010) Preferred reporting items for systematic reviews and meta-analyses: the PRISMA statement. Int J Surg 8: 336–341. 20171303 10.1016/j.ijsu.2010.02.007

[18] Higgins J, Thompson SG, Deeks JJ, Altman DG (2003) Measuring inconsistency in meta-analyses. BMJ 327: 557–560. 12958120 10.1136/bmj.327.7414.557 PMC192859

[19] Higgins JP, Thompson SG (2002) Quantifying heterogeneity in a meta-analysis. Stat Med 21: 1539–1558. 12111919 10.1002/sim.1186

[20] Higgins JPT, Green S (2008) Cochrane handbook for systematic reviews of interventions Version 5.0.0 updated February 2008. The Cochrane Collaboration.

[21] Ogino S, Nosho K, Kirkner GJ, Kawasaki T, Chan AT, et al (2012) A cohort study of tumoral LINE-1 hypomethylation and prognosis in colon cancer. J Natl Cancer Inst 100: 1734–1738. 10.1093/jnci/djn359 PMC2639290 19033568

[22] Sigalotti L, Fratta E, Bidoli E, Covre A, Parisi G, et al (2011) Methylation levels of the “long interspersed nucleotide element-1” repetitive sequences predict survival of melanoma patients. J Transl Med 9: 78. 21615918 10.1186/1479-5876-9-78 PMC3123580

[23] Bae JM, Shin SH, Kwon HJ, Park SY, Kook M, et al (2012) ALU and LINE-1 hypomethylations in multistep gastric carcinogenesis and their prognostic implications. Int J Cancer 131: 1323–1331. 22120154 10.1002/ijc.27369

[24] Van Hoesel A, van de Velde C, Kuppen P, Liefers G, Putter H, et al (2012) Hypomethylation of LINE-1 in primary tumor has poor prognosis in young breast cancer patients: a retrospective cohort study. Breast Cancer Res Treat 134: 1103–1114. 22476853 10.1007/s10549-012-2038-0

[25] Gao XD, Qu JH, Chang XJ, Lu YY, Bai WL, et al (2013) Hypomethylation of long interspersed nuclear element-1 promoter is associated with poor outcomes for curative resected hepatocellular carcinoma. Liver Int 34: 136–146. 10.1111/liv.12264 PMC4238827 23875825

[26] Iwagami S, Baba Y, Watanabe M, Shigaki H, Miyake K, et al (2013) LINE-1 Hypomethylation Is Associated With a Poor Prognosis Among Patients With Curatively Resected Esophageal Squamous Cell Carcinoma. Ann Surg 257(3): 449–55. 23023202 10.1097/SLA.0b013e31826d8602

[27] Shigaki H, Baba Y, Watanabe M, Murata A, Iwagami S, et al (2013) LINE-1 hypomethylation in gastric cancer, detected by bisulfite pyrosequencing, is associated with poor prognosis. Gastric Cancer 16: 480–487. 23179365 10.1007/s10120-012-0209-7 PMC3824342

[28] Antelo M, Balaguer F, Shia J, Shen Y, Hur K, et al (2012) A High Degree of LINE-1 Hypomethylation Is a Unique Feature of Early-Onset Colorectal Cancer. PLoS ONE 7: e45357. 23049789 10.1371/journal.pone.0045357 PMC3458035

[29] Estecio MRH, Gharibyan V, Shen L, Ibrahim AEK, Doshi K, et al (2007) LINE-1 Hypomethylation in Cancer Is Highly Variable and Inversely Correlated with Microsatellite Instability. PLoS ONE 2: e399. 17476321 10.1371/journal.pone.0000399 PMC1851990

[30] Hur K, Cejas P, Feliu J, Moreno-Rubio J, Burgos E, et al (2014) Hypomethylation of long interspersed nuclear element-1 (LINE-1) leads to activation of protooncogenes in human colorectal cancer metastasis. Gut 63: 635–646. 23704319 10.1136/gutjnl-2012-304219 PMC3884067

[31] Matsunoki A, Kawakami K, Kotake M, Kaneko M, Kitamura H, et al (2012) LINE-1 methylation shows little intra-patient heterogeneity in primary and synchronous metastatic colorectal cancer. BMC Cancer 12: 574. 23216958 10.1186/1471-2407-12-574 PMC3534591

[32] Pavicic W, Joensuu E, Nieminen T, Peltomäki P (2012) LINE-1 hypomethylation in familial and sporadic cancer. J Mol Med 90: 827–835. 22228215 10.1007/s00109-011-0854-z PMC3383956

[33] Sunami E, de Maat M, Vu A, Turner RR, Hoon DSB (2011) LINE-1 Hypomethylation During Primary Colon Cancer Progression. PLoS ONE 6(4): e18884. 21533144 10.1371/journal.pone.0018884 PMC3077413

[34] Chalitchagorn K, Shuangshoti S, Hourpai N, Kongruttanachok N, Tangkijvanich P, et al (2004) Distinctive pattern of LINE-1 methylation level in normal tissues and the association with carcinogenesis. Oncogene 23: 8841–8846. 15480421 10.1038/sj.onc.1208137

[35] Hou L, Wang H, Sartori S, Gawron A, Lissowska J, et al (2010) Blood leukocyte DNA hypomethylation and gastric cancer risk in a high-risk Polish population. Int J Cancer 127: 1866–1874. 20099281 10.1002/ijc.25190 PMC3009461

[36] Gao Y, Baccarelli A, Shu XO, Ji BT, Yu K, et al (2012) Blood leukocyte Alu and LINE-1 methylation and gastric cancer risk in the Shanghai Women's Health Study. Br J Cancer 106: 585–391. 22173668 10.1038/bjc.2011.562 PMC3273339

[37] Lee JR, Chung WC, Kim JD, Lee KM, Paik CN, et al (2011) Differential LINE-1 Hypomethylation of Gastric Low-Grade Dysplasia from High Grade Dysplasia and Intramucosal Cancer. Gut Liver 5: 149–153. 21814593 10.5009/gnl.2011.5.2.149 PMC3140658

[38] Ramzy I, Omran D, Hamad O, Shaker O, Abboud A (2011) Evaluation of serum LINE-1 hypomethylation as a prognostic marker for hepatocellular carcinoma. Arab J Gastroenterol 12: 139–142. 22055592 10.1016/j.ajg.2011.07.002

[39] Wu HC, Wang Q, Yang HI, Tsai WY, Chen CJ, et al (2012) Global DNA methylation levels in white blood cells as a biomarker for hepatocellular carcinoma risk: a nested case–control study. Carcinogenesis 33: 1340–1345. 22581841 10.1093/carcin/bgs160 PMC3499052

[40] Tangkijvanich P, Hourpai N, Rattanatanyong P, Wisedopas N, Mahachai V, et al (2007) Serum LINE-1 hypomethylation as a potential prognostic marker for hepatocellular carcinoma. Clin Chim Acta 379: 127–133. 17303099 10.1016/j.cca.2006.12.029

[41] Di JZ, Han XD, Gu WY, Wang Y, Zheng Q, et al (2011) Association of hypomethylation of LINE-1 repetitive element in blood leukocyte DNA with an increased risk of hepatocellular carcinoma. J Zhejiang Univ-Sci B 12: 805–811. 21960343 10.1631/jzus.B1000422 PMC3190095

[42] Kreimer U, Schulz WA, Koch A, Niegisch G, Goering W (2013) HERV-K and LINE-1 DNA methylation and reexpression in urothelial carcinoma. Frontiers in Oncology 3: 255. 24133654 10.3389/fonc.2013.00255 PMC3783855

[43] Wolff EM, Byun HM, Han HF, Sharma S, Nichols PW, et al (2010) Hypomethylation of a LINE-1 Promoter Activates an Alternate Transcript of the MET Oncogene in Bladders with Cancer. PLoS Genet 6: e1000917. 20421991 10.1371/journal.pgen.1000917 PMC2858672

[44] Subbalekha K, Pimkhaokham A, Pavasant P, Chindavijak S, Phokaew C, et al (2009) Detection of LINE-1s hypomethylation in oral rinses of oral squamous cell carcinoma patients. Oral Oncol 45: 184–191. 18715815 10.1016/j.oraloncology.2008.05.002

[45] Phokaew C, Kowudtitham S, Subbalekha K, Shuangshoti S, Mutirangura A (2008) LINE-1 methylation patterns of different loci in normal and cancerous cells. Nucleic Acids Res 36: 5704–5712. 18776216 10.1093/nar/gkn571 PMC2553567

[46] Pobsook T, Subbalekha K, Sannikorn P, Mutirangura A (2011) Improved measurement of LINE-1 sequence methylation for cancer detection. Clin Chim Acta 412: ; 314–321. 10.1016/j.cca.2010.10.030 21078311

[47] Daskalos A, Nikolaidis G, Xinarianos G, Savvari P, Cassidy A, et al (2009) Hypomethylation of retrotransposable elements correlates with genomic instability in non-small cell lung cancer. Int J Cancer 124: 81–87. 18823011 10.1002/ijc.23849

[48] Saito K, Kawakami K, Matsumoto I, Oda M, Watanabe G, et al (2010) Long Interspersed Nuclear Element 1 Hypomethylation Is a Marker of Poor Prognosis in Stage IA Non–Small Cell Lung Cancer. Clin Cancer Res 16: 2418–2426. 20371677 10.1158/1078-0432.CCR-09-2819

[49] Choi JY, James S, Link P, McCann S, Hong C, et al (2009) Association between global DNA hypomethylation in leukocytes and risk of breast Cancer. Carcinogenesis 30: 1889–1897. 19584139 10.1093/carcin/bgp143 PMC2783000

[50] Liao LM, Brennan P, van Bemmel DM, Zaridze D, Matveev V, et al (2011) LINE-1 Methylation Levels in Leukocyte DNA and Risk of Renal Cell Cancer. PLoS ONE 6: e27361. 22076155 10.1371/journal.pone.0027361 PMC3208631

[51] Yegnasubramanian S, Haffner M, Zhang Y, Gurel B, Cornish TC, et al (2008) DNA Hypomethylation Arises Later in Prostate Cancer Progression than CpG Island Hypermethylation and Contributes to Metastatic Tumor Heterogeneity. Cancer Res 68: 8954–67. 18974140 10.1158/0008-5472.CAN-07-6088 PMC2577392

[52] Choi IS, Estecio M, Nagano Y, Kim DH, White J, et al (2007) Hypomethylation of LINE-1 and Alu in well-differentiated neuroendocrine tumors (pancreatic endocrine tumors and carcinoid tumors). Modern Pathology 20: 802–810. 17483816 10.1038/modpathol.3800825

[53] Dammann RH, Kirsch S, Schagdarsurengin U, Dansranjavin T, Gradhand E, et al (2010) Frequent aberrant methylation of the imprinted IGF2/H19locus and LINE1 hypomethylation in ovarian carcinoma. Int J Oncol 36: 171–179. 19956846

[54] Lee JJ, Geli J, Larsson C, Wallin G, Karimi M, et al (2008) Gene-specific promoter hypermethylation without global hypomethylation in follicular thyroid cancer. Int J Oncol 33: 861–869. 18813801

[55] Shuangshoti S, Hourpai N, Pumsuk U, Mutirangura A (2007) Line-1 Hypomethylation in Multistage Carcinogenesis of the Uterine Cervix. Asian Pac J Cancer Prev 8: 307–309. 17696752

[56] Mirabello L, Savage S, Korde L, Gadalla SM, Greene MH (2010) LINE-1 methylation is inherited in familial testicular cancer kindreds. BMC Med Genet 11: 77. 20478068 10.1186/1471-2350-11-77 PMC2880977

[57] Fabris S, Bollati V, Agnelli L, Morabito F, Motta V, et al (2011) Biological and clinical relevance of quantitative global methylation of repetitive DNA sequences in chronic lymphocytic leukemia. Epigenetics 6: 188–194. 20930513 10.4161/epi.6.2.13528 PMC3775884

[58] Bollati V, Fabris S, Pegoraro V, Ronchetti D, Mosca L, et al (2009) Differential repetitive DNA methylation in multiple myeloma molecular subgroups. Carcinogenesis 30: 1330–1335. 19531770 10.1093/carcin/bgp149

[59] Geli J, Kiss N, Karimi M, Lee JJ, Backdahl M, et al (2008) Global and Regional CpG Methylation in Pheochromocytomas and Abdominal Paragangliomas: Association to Malignant Behavior. Clin Cancer Res 14: 2551–2559. 18451216 10.1158/1078-0432.CCR-07-1867

[60] Trankenschuh W, Puls F, Christgen M, Albat C, Heim A, et al (2010) Frequent and Distinct Aberrations of DNA Methylation Patterns in Fibrolamellar Carcinoma of the Liver. PLoS ONE 5: e13688. 21060828 10.1371/journal.pone.0013688 PMC2966398

[61] Igarashi S, Suzuki H, Niinuma T, Shimizu H, Nojima M, et al (2010) Novel Correlation between LINE-1 Hypomethylation and the Malignancy of Gastrointestinal Stromal Tumors. Clin Cancer Res 16: 5114–5123. 20978145 10.1158/1078-0432.CCR-10-0581

[62] Feinberg AP, Vogelstein B (1983) Hypomethylation distinguishes genes of some human cancers from their normal counterparts. Nature 301: 89–92. 6185846 10.1038/301089a0

[63] Zhu ZZ, Hou L, Bollati V, Tarantini L, Marinelli B, et al (2010) Predictors of global methylation levels in blood DNA of healthy subjects: a combined analysis. Int J Epidemiol 41: 126–139. 20846947 10.1093/ije/dyq154 PMC3304518

[64] Piyathilake CJ, Macaluso M, Alvarez RD, Chen M, Badiga S, et al (2011) A higher degree of LINE-1 methylation in peripheral blood mononuclear cells, a one-carbon nutrient related epigenetic alteration, is associated with a lower risk of developing cervical intraepithelial neoplasia. Nutrition 27: 513–59. 21463750 10.1016/j.nut.2010.08.018 PMC3070926

[65] van Bemmel D, Lenz P, Liao LM, Baris D, Sternberg LR, et al (2012) Correlation of LINE-1 methylation levels in patient matched buffy coat, serum, buccal cell and bladder tumor tissue DNA samples. Cancer Epidemiol Biomarkers Prev 21: 1143–1148. 22539607 10.1158/1055-9965.EPI-11-1030 PMC3397796

[66] Suter CM, Martin DI, Ward RL (2004) Hypomethylation of L1 retrotransposons in colorectal cancer and adjacent normal tissue. Int J Colorectal Dis 19: 95–101. 14534800 10.1007/s00384-003-0539-3

[67] Rakyan VK, Down TA, Balding DJ, Beck S (2011) Epigenome-wide association studies for common human diseases. Nat Rev Genet 12: 529–541. 21747404 10.1038/nrg3000 PMC3508712

[68] Beck S, Rakyan VK (2008) The methylome: approaches for global DNA methylation profiling. Trends Genet 24: 231–237. 18325624 10.1016/j.tig.2008.01.006

[69] Nelson H, Marsit C, Kelsey K (2011) Global Methylation in Exposure Biology and Translational Medical Science. Environ Health Perspect 119: 1528–1533. 21669556 10.1289/ehp.1103423 PMC3226501

